# Evaluation of Trophic Structure and Energy Flow in a *Pelteobagrus fulvidraco* Integrated Multi-Trophic Aquaculture System

**DOI:** 10.3390/ijerph191912027

**Published:** 2022-09-23

**Authors:** Yuxi Zhao, Xingguo Liu, Ming Lu, Runfeng Zhou, Zhaoyun Sun, Shuwen Xiao

**Affiliations:** 1 Key Laboratory of Aquaculture Facilities Engineering, Ministry of Agriculture and Rural Affairs, 63 Chifeng Road, Shanghai 200092, China; 2 Fishery Machinery and Instrument Research Institute, Chinese Academy of Fishery Sciences, 63 Chifeng Road, Shanghai 200092, China; 3 College of Fisheries and Life Science, Shanghai Ocean University, 999 Huchenghuan Road, Shanghai 201306, China; 4 College of Environment and Architecture, University of Shanghai for Science and Technology, 516 Jungong Road, Shanghai 200093, China; 5 Wuxi Fisheries College, Nanjing Agriculture University, 69 Renbin Road, Wuxi 214128, China

**Keywords:** *Pelteobagrus fulvidraco*, integrated multi-trophic aquaculture, Ecopath model, nutrient structure, energy throughput

## Abstract

An integrated multi-trophic aquaculture system (IMTA) combined muti-trophic organism cultivation with ecological engineering facilities effectively improves energy utilization efficiency and reduces pollution emission, which promotes the development of the aquaculture industry. In this study, an Ecopath model was used to analyze the *Pelteobagrus fulvidraco*-integrated multi-trophic aquaculture system (FMRP). The results showed that the effective trophic level range of FMRP was low (1~2.566), and the energy throughput was mainly concentrated in trophic level I (65.39%). The utilization rate of commercial fish feed was high. Due to the lack of predators for detritus and primary producers (*Oryza sativa* L. and hydrophyte), the energy throughput of detritus and the primary production were not fully utilized. The ascendency/total development capacity (A/TDC) and overhead/total development capacity (O/TDC) were 0.29 and 0.59, respectively, which indicated that the aquaculture system had high elasticity and strong anti-perturbation ability, but the stability could be substantially improved. The results of the carrying capacity assessment showed that the maximal single increments of *Pelteobagrus fulvidraco* fry and juvenile were 0.12 g/m^2^ and 0.42 g/m^2^, respectively, and the maximal common increments of *Pelteobagrus fulvidraco* fry and juvenile were 0.10 g/m^2^ and 0.10 g/m^2^, respectively, which indicated that there was insufficient space for increment. The study showed that the FMRP still needed to be improved in the aspects of polyculture species, energy consumption and stability. It would be necessary for the FMRP to perform further optimization and enhancement on the energy utilization efficiency, system stability and comprehensive benefits.

## 1. Introduction

As an important freshwater economic fish in China, the output of *Pelteobagrus fulvidraco* reached 565,477 tons in 2021 [1], which was a rapid development. However, for a long time, *Pelteobagrus fulvidraco* aquaculture relies on traditional aquaculture methods, which unilaterally pursues yield with excessive aquaculture density [2], thus, resulting in serious pollution [3], frequent diseases [4] and declined aquaculture quality [5]. Therefore, understanding how to build an efficient ecological aquaculture system of *Pelteobagrus fulvidraco* and improve economic and ecological benefits has become the focus and difficulty of *Pelteobagrus fulvidraco* aquaculture research.

Integrated multi-trophic aquaculture (IMTA) is an aquaculture technology that integrates ecological engineering facilities and ecological engineering systems. It sets up aquaculture facilities according to aquaculture needs and cultivates organisms in different ecological niches, which improves resource utilization efficiency and reduces water pollution [6]. Since 2000, IMTAs had been widely used in China. In East China, Central China and South China, an IMTA for *Cyprinus carpio* saved 60% of water and reduced emissions by 80% [7]. In the saline-alkali areas of Northwest and Central China, an IMTA for *Cyprinus carpio*, *Penaeus vannamei* and *Eriocheir sinensis* increased economic and ecological benefits by more than 50% [8]. In the lakeside buffer zones such as Taihu Lake, Gehu Lake and Weishan Lake, an IMTA for *Eriocheir sinensis* larvae, *Eriocheir* sinensis juvenile and herbivorous fish increased the reuse rates of nitrogen and phosphorus by more than 50% [9]. Compared with traditional aquaculture systems, IMTAs relieve the pressure of aquaculture on environment and gain diversified benefits through reasonable polyculture. Although the integrated multi-trophic aquaculture of *Pelteobagrus fulvidraco* is a new direction to promote industrial upgrading, few studies have been performed.

In IMTAs, the difference in species and quantity of polyculture organisms is the main factor leading to the difference in aquaculture system functions. Therefore, exploring the nutrient structure and energy characteristics is key to build an efficient IMTA. The Ecopath model is a nutrient balance model, based on the principle of nutrient dynamics, describing energy throughput and the material cycle in the ecosystem [6]. As the core tool for studying the aquatic ecosystem [10], the Ecopath model has been used to study keystone species, trophic structure and energy transfer efficiency of natural [11] and aquaculture water bodies [12,13,14,15,16]. Applying the Ecopath model to analyze the *Pelteobagrus fulvidraco* aquaculture system will provide a reference for improvement.

In this study, a *Pelteobagrus fulvidraco*-integrated multi-trophic aquaculture system (FMRP) was constructed and an Ecopath model was developed. The specific objectives were as follows: (i) to simulate the nutrient structure and energy throughput process of the FMRP; (ii) to analyze the ecological characteristics of the FMRP quantitatively; (iii) to evaluate the carrying capacity of *Pelteobagrus fulvidraco* preliminarily. It will provide a theoretical basis for optimizing the FMRP and promoting the sustainable development of the *Pelteobagrus fulvidraco* aquaculture industry.

## 2. Materials and Methods

### 2.1. Construction of FMRP

**Figure 1:**
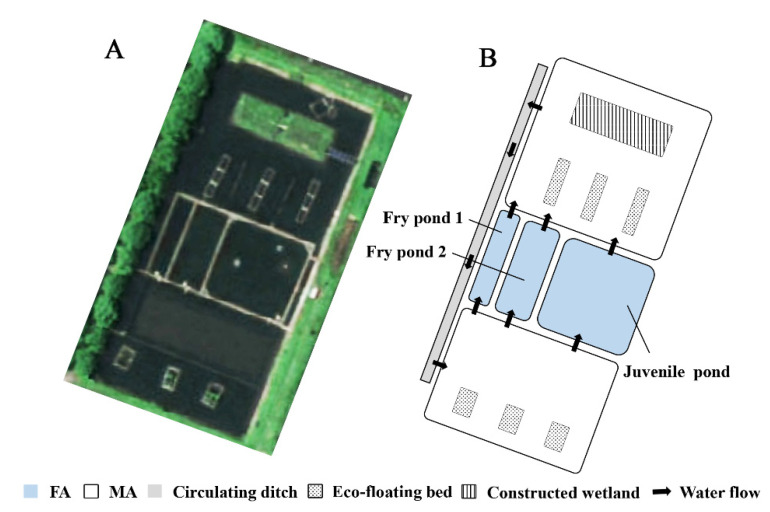
The actual picture (**A**) and structural layout (**B**) of the FMRP.

The experiment was carried out in Songjiang farming base (30°95′ N, 121°16′ E), Fishery Machinery and Instrument Research Institute, Chinese Academy of Fishery Sciences, Shanghai, China. The experiment lasted for 150 days from April 2021 to August 2021. The experimental pond area was 0.48 ha, and the average water depth was 1.4 m. In order to achieve the purpose of polyculture and promote the growth of different organisms, the FMRP separated organisms from different niches by dividing the pond into a *Pelteobagrus fulvidraco* aquaculture area (FA) and a mixed polyculture area (MA), which were connected by a circulating water system [17]. In the FA, fry pond 1, fry pond 2 and a juvenile pond were arranged in parallel to cultivate *Pelteobagrus fulvidraco* with different specifications to minimize predation. The structure of the FMRP is shown in Figure 1.

Referring to the species of various trophic levels in the natural habitat of *Pelteobagrus fulvidraco* [18,19,20] and other IMTA [21], organisms in different trophic levels were cultivated in the FMRP according to ecological niche differences. As for animals, *Pelteobagrus* *fulvidraco* fries were stocked in the FA fry pond with an initial weight of 0.375 g and stocking density of 2.10 ind/m^2^; *Pelteobagrus fulvidraco* juveniles were stocked in the juvenile pond with an initial weight of 18.92 g and a stocking density of 0.63 ind/m^2^; crustaceans, including *Macrobrachium nipponense*, *Procambarus clarkii*, *Eriocheir sinensis* and *Bellamya aeruginosa*, were stocked in the MA with initial body weights of 1.93 g, 15.66 g, 3.57 g and 2.12 g, respectively, and stocking densities of 1.09 ind/m^2^, 2.15 ind/m^2^, 0.21 ind/m^2^ and 23.16 ind/m^2^, respectively. In terms of plants, cash crop (*Oryza sativa* L.) was planted in the MA, with the initial planting density of 10.44 g/m^2^; hydrophytes were planted in the MA with total initial planting density of 305.28 g/m^2^, including *Ipomoea aquatica* Forsk, *Hydrocharis dubia* (Bl.) Backer, *Cyperus invucratus* Rottboll and *Vallisneria natans* (Lour.) Hara.

### 2.2. Culture Management

During the experiment, commercial fish feed containing 40% crude protein, 6% crude fiber and 5% lipid (Tongwei Co., Ltd., Wuxi, China) was provided in the FA at 8:00 a.m. and 17:00 p.m. every day, but no feeding or fertilization was given in the MA. The feeding amount was recorded every day. In order to ensure the healthy growth of stocking organisms, 15~30 fishes, shrimps and crabs were trapped in cages (0.4 m × 0.4 m × 2 m) every 30 days. After the inspection was completed, the sampled organisms were put back into the sampling site.

### 2.3. Construction of Ecopath Model

In the Ecopath model, the ecosystem was divided into related functional groups. According to the principle of energy conservation, a set of simultaneous linear equations was established to balance the energy input and output in each functional group (reduction-predator mortality–harvest-net migration-bioaccumulation = 0), thus obtaining the static balance model of the ecosystem at a specific time, which could be simplified as [22]:

(1) $$B_{i}\cdot {\left(\frac{P}{B}\right)}_{i}{\cdot EE}_{i}-\sum_{j}B_{j}\cdot {\left(\frac{Q}{B}\right)}_{j}{\cdot DC}_{ij}-Y_{i}-{BA}_{i}-E_{i}=0$$

where $B_{i}$ represents prey biomass, $B_{j}$ represents predator biomass, (*P/B*)*_i_* represents the ratio of prey production to biomass, ${EE}_{i}$ represents ecotrophic efficiency, (*Q/B*)*_j_* is the ratio of predator food consumption to biomass, ${DC}_{ij}$ represents the ratio of prey *i* to predator *j*’s total food consumption, $Y_{i}$ represents catch, ${BA}_{i}$ represents prey *i*’s bioaccumulation and $E_{i}$ represents the net migration rate (difference between emigration and immigration).

### 2.4. Functional Group Settings

According to the definition and setting principle of functional groups in the Ecopath model, different organisms in the FMRP were divided into independent functional groups according to feeding habits, ecological functions, economic values and growth stages [23,24,25]. There were 16 functional groups in the model, including *Pelteobagrus fulvidraco* fry, *Pelteobagrus fulvidraco* juvenile, *Pseudorasbora parva*, *Macrobrachium nipponense*, *Procambarus clarkii*, *Eriocheir sinensis*, *Bellamya aeruginosa*, Copepoda, Cladocera, Rotifera, bacteria, *Oryza sativa* L., hydrophyte, phytoplankton, commercial fish feed and detritus. Because commercial fish feed only provides energy for the system, this functional group was defined as a detritus functional group in the model [22]. In addition, another detritus function group was set up to analyze the organic detritus in the aquaculture system. Because there were plural detritus functional groups in the model, it was necessary to describe the proportion of each functional group that flowed to different detritus functional groups [26]. In this experiment, all the unused parts of each functional group were set to flow into the detritus functional group of detritus.

### 2.5. Model Parameters and Data Collection

The main input parameters of the Ecopath model included biomass (*B*), production/biomass (*P*/*B*), consumption /biomass (*Q*/*B*) and diet composition in each functional group. The biomass was expressed as g/m^2^. The biomass of commercial fish feed was calculated as the average of daily inputs of the 150-day experiment. The biomass of other functional groups was determined by sampling data every 30 days and was expressed as the average of the 150-day experiment.

In the aspect of *P*/*B*, the production of *Pelteobagrus fulvidraco* (fry and juvenile), *Pseudorasbora parva*, *Macrobrachium nipponense*, *Procambarus clarkii*, *Eriocheir sinensis*, *Bellamya aeruginosa* and *Oryza sativa* L. were calculated as the differences between initial and ultimate weight, and the *P*/*B* values were calculated using production values and biomass [27,28]. Phytoplankton production was calculated by the black and white bottle method [29], and the *P*/*B* value was calculated using production values and biomass. The *P*/*B* values of hydrophyte [19], zooplankton (Cladocera, Copepoda, Rotifera) [22] and bacteria [30] referred to the related studies.

In terms of the *Q*/*B*, the *Q*/*B* values of *Pelteobagrus fulvidraco* (fry and juvenile) were calculated using the feeding amount and biomass. The *Q*/*B* values of *Pseudorasbora parva* [19], *Macrobrachium nipponense* [19], *Procambarus clarkii* [30], *Eriocheir sinensis* [20], *Bellamya aeruginosa* [31], zooplankton [22] and bacteria [30] referred to the relevant studies.

Diet composition refers to the proportion of each instance of food consumption to total food consumption in a certain organism. The diet compositions of *Pelteobagrus fulvidraco* (fry and juvenile) and *Pseudorasbora parva* were obtained by carbon isotope analysis and referred to the related studies [32,33]. The diet compositions of *Macrobrachium nipponense* [19,34,35], *Procambarus clarkii* [30,36], *Eriocheir sinensis* [20,33,35,37], *Bellamya aeruginosa* [20,33,35,38], zooplankton [22,35,39] and bacteria [30] referred to the relevant studies. The sum of the diet composition of each functional group was 1 (Table 1). In addition, the proportion of unassimilated food of zooplankton was set to 0.4, and other functional groups were set to 0.2 [6].

### 2.6. Model Balancing

In the debugging of the model, the ecotrophic efficiency (EE) value (≤1) was the basic limiting condition in each functional group. If EE > 1, this indicated that the biological consumption was higher than the production. In this case, it was necessary to fine-tune the input *P*/*B*, *Q*/*B* or food composition to make the output EE within a reasonable range [19,40] and balance the model. The pedigree index was used to evaluate the quality of the model, which evaluated the accuracy of the model by defining the source of input data and quality. When it was >0.7, the reliability of the model was high [41].

### 2.7. Carrying Capacity Assessment

The carrying capacity assessment estimates the maximum biomass limit for certain species in different ecosystems, which is helpful when adjusting the biomass of organisms. In this manuscript, we analyzed the maximum common increment (an increase in both fry and juvenile biomass) and the maximum single increment (an increase in fry and juvenile biomass, respectively) of *Pelteobagrus fulvidraco* based on the Ecopath model. By increasing the biomass continuously (0.02 g/m^2^ each time) until EE > 1 in any functional group, the biomass of *Pelteobagrus fulvidraco* reached the carrying capacity [19,30,42].

### 2.8. Ecosystem Analysis Derived from Ecopath Model Application

In this manuscript, a variety of parameters were presented to analyze the characteristics of the FMRP based on the Ecopath with Ecosim (v6.5).

The transfer efficiency (TE) is calculated as the ratio between the production of a given trophic level and the production of the previous trophic level [43]. The ecotrophic efficiency (EE) value is estimated as the part of production that is used within or exported out of the ecosystem. The total biomass (excluding detritus) (TB) is the sum of biomass of all groups except for detritus. The sum of all production (TP) refers to the total production created by all components. The sum of all consumption (TC) is the total intake of food of all consumers. The sum of all respiratory flows (TR) is the total non-usable energy leaving the ecosystem. The sum of all flows into detritus (TD) is the total energy flowing into detritus [44]. The total system throughput (TST) is the sum of all flows in a system, including TC, sum of all exports, TR and TD [45]. The total net primary production (TPP) is calculated as the summed primary production from all producers. The net system production is the difference between total primary production and total respiration [44]. 

The total development capacity (TDC) is the product of the flow diversity (diversity of interactions between ecosystem components) and the TST, which is the largest value of ascendency (A) and represents the upper limits of system development [45]. The A is the product of the average mutual information in an ecosystem and the TST [6]. The overhead (O) is the difference between the TDC and the A, which calculates the uncertainty of energy flow of the network [46] and represents the unorganized part of TDC in an ecosystem [6]. The connectance index (CI) is the ratio of the number of actual links to the number of possible links in a food web. Feeding on detritus (by detritivores) is included in the count. The system omnivory index (SOI) is defined as the average omnivory index of all consumers weighted by the logarithm of each consumer’s food intake, which is a measure of how the feeding interactions are distributed between trophic levels. The ecopath pedigree index (EPI) is the product of all the pedigree parameter specific indices from functional groups, which represents a matching degree between the data and model [44]. The Finn’s cycling index (FCI) represents the fraction of an ecosystem’s throughput recycled, correlating with the system maturity, resilience and stability. The Finn’s cycling mean path length (FCL) is defined as the average number of groups that an inflow or outflow passes through [47]. 

The mixed trophic impact (*MTI*) analysis constructs a matrix to assess the effect that changes in the biomass of a group will have on the biomass of the other groups in an ecosystem [48]. The *MTI* value is calculated as:

(2) $${MTI}_{ij}={DC}_{ij}-{\;FC}_{ji}$$

where ${MTI}_{ij}$ is the *MTI* value, ${DC}_{ij}\;$ is the diet composition term expressing how much *j* contributes to the diet of *i*,${\;FC}_{ji}$ is a host composition term giving the proportion of the predation on *j* that is due to *i* as a predator. Results are visualized as black and white dot plots in the Ecopath model to show positive and negative effects.

The keystone species is defined as a relatively low biomass species with a structuring role in their food webs [49]. The keystone index value is calculated as:

(3) $${KS}_{i}={\log[\varepsilon }_{i}\cdot (1\;-{\;P}_{i})]$$

where ${KS}_{i}$ is the keystone index of group (*i*), $\varepsilon_{i}$ is a constant which quantifies direct and indirect impacts on impacted groups from impacting groups, $P_{i}$ is the ratio of the biomass (*i*) ($B_{i}$) to the biomass of the total ecosystem. The higher the keystone index is in magnitude, the more important the species is.

The Lindeman spine analysis aggregates the entire system into discrete trophic levels and shows the distribution of energy flows. The ratio of energy in different trophic levels is presented clearly [44].

## 3. Results

### 3.1. Parameter Estimation of the Ecopath Model

After inputting the relevant parameters and balancing the model, the output results were obtained (Table 2). *Pelteobagrus fulvidraco* fry, *Pelteobagrus fulvidraco* juvenile and *Oryza sativa* L. showed high EE values, which were 0.988, 0.980 and 0.991, respectively. The EE values of commercial fish feed, phytoplankton, Copepoda and Cladocera were 0.997, 0.931, 0.856 and 0.876, respectively, which indicated a high utilization rate. The EE value of bacteria was 0.123, indicating that the utilization rate of bacteria was lowest in the FMRP.

### 3.2. Features of Food Web

**Figure 2:**
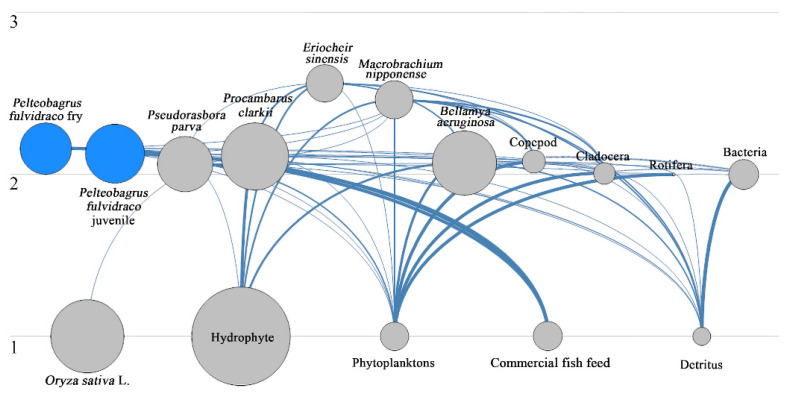
Trophic structure of the FMRP. Circles represent functional groups, the size of a circle represents biomass size, numbers represent effective trophic levels, and the lines represent the direction of energy flow and predation relationship.

By dividing integrated trophic levels and calculating the effective trophic level (ETL) in each functional group according to the energy throughput ratio and integrated trophic level in each functional group, the complex food web relationship in ecosystems can be simplified and the trophic status of each functional group can be displayed intuitively. In the FMRP, the effective trophic levels ranged from 1 to 2.566. *Oryza sativa* L., hydrophyte, phytoplankton, commercial fish feed and detritus were in trophic level Ⅰ. *Eriocheir sinensis* and *Macrobrachium nipponense* were between trophic level Ⅱ and Ⅲ. Other functional groups belonged to trophic level II (Figure 2). *Eriocheir sinensis* was in the highest effective trophic level. The energy throughput of the system was mainly concentrated in trophic levels I and II (Table 2).

### 3.3. System Characteristics

The analysis of overall characteristics can intuitively reflect the scale, stability and maturity of the ecosystem. The TST of the FMRP was 6626.25 g/m^2^·150 days. The TC, TR and TD were 2315.04, 919.21 and 1914.74 g/m^2^·150 days, accounting for 34.94%, 13.87% and 28.90% of the TST, respectively. Food consumption was the largest component of TST. The TPP and TPP/TR were 2832.87 g/m^2^·150 days and 3.08, respectively. The TPP was higher than the TR, which indicated that the primary production capacity of FMRP was high. The O/TDC and SOI were 0.59 and 0.11, respectively. The A/TDC and CI were 0.29 and 0.28, respectively. The FCI and FCL were 20.52% and 2.77, respectively. The EPI was 0.78, which was higher than the numerical range of 0.164~0.676 in 393 Ecopath models calculated by Morissett [50], which showed that the model had high reliability (Table 3).

### 3.4. Energy Consumption by Consumers

**Figure 3:**
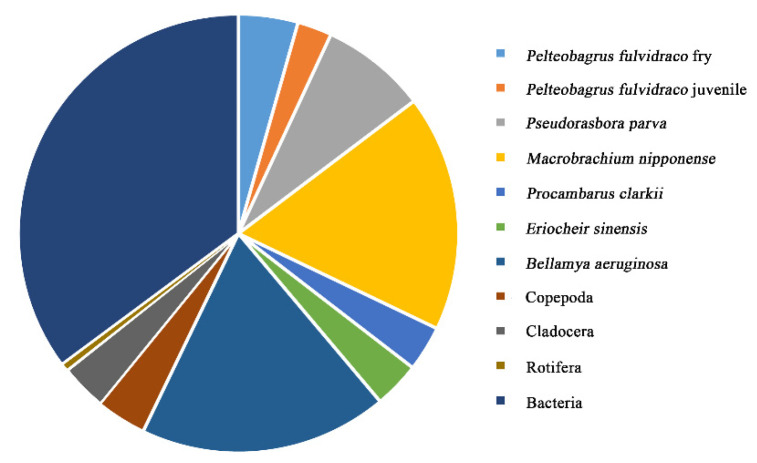
Proportion of energy throughput consumption.

In the FMRP, bacteria were the largest consumer, consuming 35.18% of energy throughput of the TC. The energy throughput consumption ratios of *Pelteobagrus fulvidraco* (fry and juvenile) were 4.37% and 2.53%, respectively, accounting for 6.80% in total. The energy throughput consumption ratios of other species (*Pseudorasbora parva*, *Macrobrachium nipponense*, *Procambarus clarkii*, *Eriocheir sinensis* and *Bellamya aeruginosa*) accounted for 50.22% in total. The energy throughput consumption ratio of zooplankton (Copepoda, Cladocera and Rotifera) accounted for 7.69% in total. The results showed that the energy throughput consumption of *Pelteobagrus fulvidraco* was low (Figure 3).

### 3.5. Characteristics of Energy Conversion

**Figure 4:**
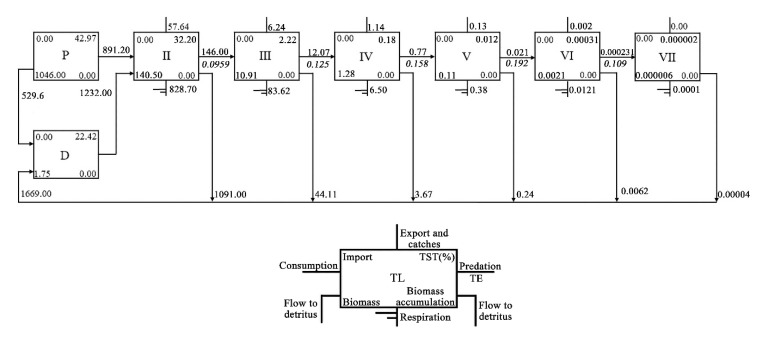
Energy flows among different trophic levels in the culture system. P: primary producer; D: detritus.

Lindeman spine analysis is an effective way to determine the energy transfer efficiency between trophic levels [20,51]. In the FMRP, the energy throughput of trophic level Ⅰ and Ⅱ accounted for 65.39% and 32.20% of the TST, respectively. The TE of trophic level Ⅰ was 49.25%, which was highest. The TE of trophic level Ⅱ was 9.59%. The inflow energy throughput of detritus was 2198.60 g/m^2^·150 days, of which 12,232.00 g/m^2^·150 days (56.04%) flowed into trophic level II; the rest was not utilized and instead accumulated at the bottom of the pond. (Table 4, Figure 4).

### 3.6. Analysis of Mixed Trophic Impact

**Figure 5:**
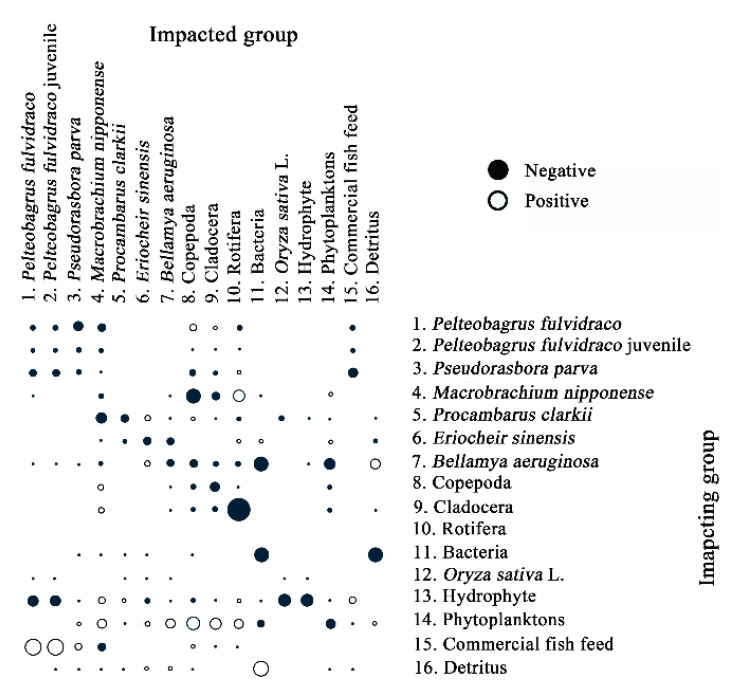
A mixed trophic impact analysis in the FMRP.

The mixed trophic impact (MTI) analysis is an effective way to analyze the direct and indirect interaction between different populations in an ecosystem [52]. All consumers in the FMRP showed a strong internal density restriction effect. Commercial fish feed could promote the *Pelteobagrus fulvidraco* (fry and juvenile) and *Pseudorasbora parva*. Phytoplankton could promote crustaceans (*Macrobrachium nipponense*, *Procambarus clarkii*, *Eriocheir sinensis* and *Bellamya aeruginosa*). This result indicated that commercial fish feed and phytoplankton played an important role in the food sources. *Pseudorasbora parva* had an inhibitory effect on *Pelteobagrus fulvidraco* (fry and juvenile), indicating a competitive relationship. Bacteria had a strong inhibitory effect on detritus, suggesting that bacteria depended on detritus as food source. *Bellamya aeruginosa* had a strong inhibitory effect on phytoplankton and bacteria. The feeding effect was obvious (Figure 5).

### 3.7. Keystone Functional Group Analysis

The keystone index value was calculated based on the Ecopath model. The higher the keystone index value was in magnitude, the more important the functional group was. Cladocera and phytoplankton were the keystone functional groups in the FMRP, and the keystone index values were −0.09 and −0.12, respectively. The keystone index values of Rotifera, bacteria and *Oryza sativa* L. were −2.11, −1.03 and −1.00, respectively, showing light influences on the ecosystem (Table 5).

### 3.8. Carrying Capacity Estimation

*Pelteobagrus fulvidraco* was the main stocking species in this experiment. By increasing the stocking amount of juveniles and fries until model unbalance, the carrying capacity of *Pelteobagrus fulvidraco* was obtained. The results showed that the maximum single increments of *Pelteobagrus fulvidraco* fries and juveniles were 0.12 and 0.42 g/m^2^, respectively, while the maximum common increments of fries and juveniles were 0.10 and 0.10 g/m^2^, respectively. The space for raising was insufficient (Table 6).

## 4. Discussion

In this study, the data came from a field investigation and related research. The EPI of the model was 0.78, which was of high reliability. The TE of FMRP (49.25%) was higher than that in traditional aquaculture systems (23.90%) [53], indicating that polyculture organisms might improve the energy utilization efficiency. The effective trophic level range (1~2.566) and trophic level I energy throughput ratio (65.39%) reflected the characteristics of concentrated energy throughput at low trophic levels and a simple trophic structure in the aquaculture system, which was consistent with the results from a shrimp–crab IMTA and a rice–crab IMTA [6,21]. Due to feed input and a lack of high trophic predators (such as carnivorous fish), the energy throughput of the aquaculture systems were mainly concentrated in low trophic levels [6]. This simple trophic structure of the FMRP might be responsible for the lower SOI, CI, A/TDC and FCI values (0.11, 0.28, 0.29, 2.77, respectively) than those of ecosystems with a complex trophic structure [54,55], representing the insufficient stability of system. However, because of this reason, the FCI value (20.41) in the FMRP was higher than that in many natural ecosystems, such as the Wuli Lake ecosystem (15.51) [33] and the Taihu Lake ecosystem (11.58) [35]. It was speculated that the high efficiency and proportion of detritus energy throughput recycling into system energy circulation [47,56] improved the energy cycle efficiency and anti-perturbation ability, which could be reflected by a high O/TDC (0.59) value [6]. Because aquaculture systems need to face the threat of external environmental changes, diseases and insect pests, resilience against external perturbations is beneficial to aquaculture activities. According to our analysis, the FMRP had an eminent anti-perturbation ability.

Compared with the traditional monoculture system, IMTAs have higher energy utilization efficiency, lower accumulation of nitrogen and phosphorus nutrients, and diversified and stable farming income. However, allocating more energy to maintain the system structure usually results in a decrease in the bioaccumulation of target farming organisms, which contradicts the goal of obtaining a high yield [57,58]. In the FMRP, the energy consumption of *Pelteobagrus fulvidraco* (fry and juvenile) (6.80% in total) was lower than that of other polyculture organisms (*Pseudorasbora parva*, *Macrobrachium nipponense*, *Procambarus clarkii*, *Eriocheir sinensis* and *Bellamya aeruginosa*) (50.22% in total), which might be related to the small culture scale of *Pelteobagrus fulvidraco* and the unreasonable biomass ratio with other polyculture organisms. In addition, the mixed trophic impact analysis showed that *Pseudorasbora parva* had an inhibitory effect on *Pelteobagrus fulvidraco*, except for the density restriction effect inside *Pelteobagrus fulvidraco.* This might be conducive to the growth of *Pelteobagrus fulvidraco* due to the competitive relationship. In terms of system energy utilization, fishing or predation pressure might be the reason for high EE values (>0.8) of *Pelteobagrus fulvidraco* (juvenile and fry), *Oryza sativa* L., zooplankton (Copepoda and Cladocera) and commercial fish feed in the FMRP. Commercial fish feed was fully utilized. As an important food source and keystone species, phytoplankton was fed upon by zooplankton, *Pseudorasbora parva* and *Macrobrachium nipponense*, which had a high EE value (0.93). It was speculated that the bottom-up effect was obvious [59]. The absence of predators and unreasonable management might be related to the low EE value of hydrophytes (0.77), because it led to the underutilization of energy. Although there were no distinct differences between the hydrophyte EE value of the FMRP and other ecosystems [31,35], the insufficient utilization of primary production energy was detrimental from the perspective of aquaculture production. It would not only reduce the production efficiency [58], but also deteriorate water quality and damage the farming environment due to late harvesting and plant decay caused by improper management. In the FMRP, only 56.04% of the detritus energy throughput flowed into the next trophic level, and the rest accumulated at the bottom of the pond underutilized. In addition to the lack of direct predators, the low utilization rate of bacterial energy might be responsible for the low detritus EE value (0.59). Bacteria were the largest consumer of detritus energy throughput in the FMRP (814.50 g/m^2^·150 days) with an EE value of 0.12. There was only *Bellamya aeruginosa* that had an obvious predation effect on bacteria. This might be because detritus energy hardly transfers to a higher trophic level, thus, impeding the detritus energy cycle [22,57].

**Figure 6:**
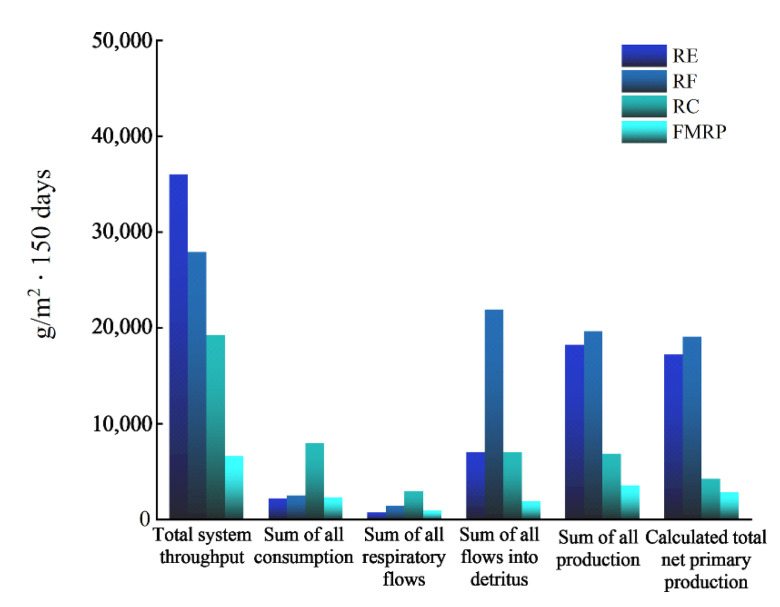
Comparison of energy flow parameters. RE: Rice–crab integrated multi-trophic aquaculture system; RF: Rice–carp integrated multi-trophic aquaculture sysytem; RC: Rice–crayfish integrated multi-trophic aquaculture system; FMRP: *Pelteobagrus fulvidraco*-integrated multi-trophic aquaculture system.

In this study, we constructed a FMRP prototype, which had a relatively complete ecological structure and diversified farming income due to planting cash crops, hydrophytes and cultivating muti-trophic organisms. At present, there were few similar *Pelteobagrus fulvidraco* aquaculture systems. However, a comparison of system characteristic parameters of the FMRP with those of other IMTAs with a similar function and structure is useful in assessing the relative performance of the FMRP. The TST (6626.25 g/m^2^·150 days), TP (3567.19 g/m^2^·150 days) and TPP (2832.87 g/m^2^ 150 days) values of FMRP were lower than those in the rice–carp IMTA, the rice–crab IMTA and the rice–crayfish IMTA [30] (Figure 6), indicating the small scale and insufficient biological resources available. The TPP/TR value (3.08) was higher than that in the Gehu Lake ecosystem (1.25) [18] and the shrimp–shellfish IMTA (2.19) [60], indicating that the FMRP might have a great development potential [57]. It may be necessary to increase the levels of biomass and feeding for scale expansion.

In order to improve the energy utilization rate and increase the income of *Pelteobagrus fulvidraco* aquaculture, the stocking amount of *Pelteobagrus fulvidraco* should be increased and the collocation of polyculture organisms should be optimized. The results of the carrying capacity assessment showed that there was insufficient increment space for *Pelteobagrus fulvidraco*, which might be related to the low feeding level and competition between *Pelteobagrus fulvidraco* and wild fishes. Thus, the feeding level should be increased, and wild fishes should be removed in time to prevent feeding competition. Based on this, the energy consumption ratio of *Pelteobagrus fulvidraco* will probably increase through raising *Pelteobagrus fulvidraco* and reducing other polyculture species appropriately for more farming income. It may be useful to reduce the water exchange rate in the FMRP to provide abundant food sources for polyculture organisms by increasing phytoplankton biomass [61]. In view of the low energy utilization rate of hydrophyte and detritus, it is likely to strengthen the energy utilization of primary producers, detritus and microorganisms by enriching cash crops, timely harvesting, culturing herbivorous organisms (such as grass carp), filter-feeding organisms (such as silver carp and bighead carp) and increasing benthic organisms (such as snails). The specific polyculture species and effects still need to be further explored.

## 5. Conclusions

This study clarified the characteristics of energy flow and trophic structure in the FMRP. The results showed that the FMRP had high commercial fish feed utilization efficiency, transfer efficiency and anti-perturbation ability, which was beneficial to the aquaculture activities. However, the small culture scale and simple trophic structure caused many problems, such as the insufficient utilization of detritus, primary production energy and low energy consumption ratio of *Pelteobagrus fulvidraco*. Optimizing polyculture collocation, adjusting the feeding strategy and improving aquaculture management would be effective for structure improvement, stability enhancement and increasing of benefits.

In an effort to fully implement green ecological aquaculture, the Ecopath technique can provide theoretical guidance for the transformation and upgrading of *Pelteobagrus fulvidraco* aquaculture.

## Figures and Tables

**Table 1:** Diet composition of the FMRP.

Prey	Predator										
	1	2	3	4	5	6	7	8	9	10	11
1 *Pelteobagrus fulvidraco* fry											
2 *Pelteobagrus fulvidraco* juvenile											
3 *Pseudorasbora parva*	0.0900	0.0700	0.0010			0.0080					
4 *Macrobrachium nipponense*	0.0400	0.0300			0.0110						
5 *Procambarus clarkii*					0.0770	0.2500					
6 *Eriocheir sinensis*						0.0200					
7 *Bellamya aeruginosa*						0.1800					
8 Copepoda	0.0033	0.0040	0.0320	0.2350	0.0060	0.0300					
9 Cladocera	0.0042	0.0032	0.0280	0.2040	0.0030	0.0200		0.0750			
10 Rotifera								0.0018	0.0086		
11 Bacteria							0.0700	0.0900	0.0290	0.0370	
12 *Oryza sativa* L.					0.1170						
13 Hydrophyte			0.0320	0.2400	0.6120	0.2600	0.2800				
14 Phytoplankton	0.0008	0.0008	0.152	0.1700	0.06700	0.0900	0.5000	0.8020	0.8510	0.9130	
15 Commercial fish feed	0.8000	0.8200	0.641								
16 Detritus	0.0621	0.0720	0.1140	0.1510	0.1070	0.1420	0.1500	0.0310	0.1110	0.0500	1.0000
Sum	1.0000	1.0000	1.0000	1.0000	1.0000	1.0000	1.0000	1.0000	1.0000	1.0000	1.0000

Values are the proportion of the prey in the food composition of the predator.

**Table 2:** Basic estimates of the Ecopath model.

Functional Groups	Biomass (g/m^2^)	*P*/*B* (150 Days)	*Q*/*B* (150 Days)	Feed Import (g/m^2^·150 Days)	Ecotrophic Efficiency	Effective Trophic Level
1 *Pelteobagrus fulvidraco* fry	12.17	1.96	8.31		0.988	2.164
2 *Pelteobagrus fulvidraco* juvenile	24.73	1.55	2.37		0.980	2.127
3 *Pseudorasbora parva*	16.38	2.25	11.00		0.489	2.068
4 *Macrobrachium nipponense*	3.23	4.50	24.40		0.705	2.487
5 *Procambarus clarkii*	50.46	3.24	8.00		0.309	2.112
6 *Eriocheir sinensis*	3.13	2.46	24.74		0.201	2.566
7 *Bellamya aeruginosa*	39.84	1.33	10.61		0.263	2.070
8 Copepoda	0.77	48.00	120.00		0.856	2.170
9 Cladocera	0.62	57.00	143.00		0.876	2.038
10 Rotifera	0.01	117.00	293.00		0.785	2.037
11 Bacteria	1.50	217.00	543.00		0.123	2.000
12 *Oryza sativa* L.	92.26	2.42			0.991	1.000
13 Hydrophyte	952.70	2.25			0.769	1.000
14 Phytoplankton	1.27	367.19			0.931	1.000
15 Commercial fish feed	1.33			245.30	0.997	1.000
16 Detritus	0.42				0.592	1.000

**Table 3:** Systematic characteristics.

Parameter		Unit
Total system throughput (TST)	6626.25	g/m^2^·150 days
Sum of all consumption (TC)	2315.04	g/m^2^·150 days
Sum of all respiratory flows (TR)	919.22	g/m^2^·150 days
Sum of all flows into detritus (TD)	1914.74	g/m^2^·150 days
Sum of all production (TP)	3567.19	g/m^2^·150 days
Total net primary production (TPP)	2832.87	g/m^2^·150 days
Net system production	1913.65	g/m^2^·150 days
Total biomass (excluding detritus) (TB)	1198.99	g/m^2^
Total primary production/Total respiration (TPP/TR)	3.08	
Total primary production/Total biomass (TPP/TB)	2.36	
Total biomass/Total throughput (TB/TP)	0.18	
Proportion of total flow originating from detritus	0.42	% of total throughput
Proportion of total flow originating from primary producers	0.58	% of total throughput
Ascendency (A)	8786.00	flowbits/m^2^·150 days
Overhead (O)	17,910.00	flowbits/m^2^·150 days
Total development capacity (TDC)	30,606.00	flowbits/m^2^·150 days
Ascendency/Total development capacity (A/TDC)	0.29	
Overhead/Total development capacity (O/TDC)	0.59	
Connectance index (CI)	0.28	
System omnivory index (SOI)	0.11	
Ecopath pedigree index (EPI)	0.78	
Finn’s cycling index (FCI, %)	20.41	% of total throughput
Finn’s cycling mean path length (FCL)	2.77	

**Table 4:** Distribution of throughput, biomass and catch in the FMRP culture system.

Trophic Level	Throughput (g/m^2^·150 Days)	Biomass (g/m^2^·150 Days)
VII	0.00014	0.0000060
VI	0.021	0.0021
V	0.77	0.11
IV	12.07	1.28
III	146.00	10.91
II	2123.00	140.50
I	4311.00	1046.00
Sum	6593.00	1187.89

Biomass in TL I excluded detritus.

**Table 5:** Overall impact and keystone indexes of the FMRP.

Functional Groups	Keystone Index
1 *Pelteobagrus fulvidraco* fry	−0.21
2 *Pelteobagrus fulvidraco* juvenile	−0.58
3 *Pseudorasbora parva*	−0.42
4 *Macrobrachium nipponense*	−0.19
5 *Procambarus clarkii*	−0.48
6 *Eriocheir sinensis*	−0.32
7 *Bellamya aeruginosa*	−0.27
8 Copepoda	−0.47
9 Cladocera	−0.09
10 Rotifera	−2.11
11 Bacteria	−1.03
12 *Oryza sativa* L.	−1.00
13 Hydrophyte	−0.79
14 Phytoplankton	−0.12

The keystone index was represented by the keystone index#1 value in Ecopath with Ecosim (v6.5).

**Table 6:** Carrying capacity assessment.

Increased Species	Increment (g/m^2^)
*Pelteobagrus fulvidraco* fry	0.12
*Pelteobagrus fulvidraco* juvenile	0.42
*Pelteobagrus fulvidraco* fry and juvenile	0.10

## Data Availability

The data that support the findings of this study are available from the corresponding author upon request.
